# Revision of fossil species of *Dryinus* belonging to *lamellatus* group, with description of a new species (Hymenoptera, Dryinidae)

**DOI:** 10.3897/zookeys.130.1335

**Published:** 2011-09-24

**Authors:** Massimo Olmi, Adalgisa Guglielmino

**Affiliations:** 1 Department of Plant Protection, University of Tuscia, Via S. Camillo de Lellis, I-01100 Viterbo, Italy

**Keywords:** Taxonomy, Dryinus rasnitsyni, amber, Dominican Republic, key, Dryinus lamellatus group, Dryininae

## Abstract

*Dryinus rasnitsyni* **sp. n.** is described from amber collected in the Dominican Republic. A revision and a key to the fossil Neotropical species of *Dryinus* Latreille, 1804 belonging to the *lamellatus* species group is presented.

## Introduction

Dryinidae (Hymenoptera: Chrysidoidea) are parasitoids of Auchenorrhyncha (Guglielmino and Olmi 1997, 2006, 2007). *Dryinus* Latreille, 1804, belonging to Dryininae, is present in all zoogeographical regions. Two hundred and seventy-eight species of *Dryinus* have been described from all over the world, of which seventeen are fossil species (Olmi 1984, 1995, 1999; Olmi and Bechly 2001; Olmi et al. 2010). According to Olmi (1993), *Dryinus* is divided into four groups: *constans*, *ruficauda*, *lamellatus* and *autumnalis*. In the *lamellatus* group only one fossil species has been described: *Dryinus grimaldii* Olmi, 1995.

In 2010 the authors have found a further new fossil species of the *lamellatus* group, which is described herein.

## Material and methods

The descriptions follow the terminology used by Olmi (1984, 1994, 1999). The measurements reported are relative, except for the total length (head to abdominal tip, without the antennae), which is expressed in millimetres. In the descriptions, POL is the distance between the inner edges of the lateral ocelli; OL is the distance between the inner edges of a lateral ocellus and the median ocellus; OOL is the distance from the outer edge of a lateral ocellus to the compound eye; OPL is the distance from the posterior edge of a lateral ocellus to the occipital carina; TL is the distance from the posterior edge of an eye to the occipital carina.

A redescription of *Dryinus grimaldii* is provided for completeness in this updated treatment of all fossil species of the *lamellatus* group.

The material studied herein is deposited in the following institutions:

AMNH American Museum of Natural History, New York (USA).

GPJC Private collection of George Poinar, Jr., c/o Department of Entomology, Oregon State University, Corvallis, Oregon (USA).

SNMS Staatliches Museum für Naturkunde Stuttgart, Abt. Paläontologie–Sektion Bernstein, Stuttgart (Germany).

## Systematics

### Genus Dryinus

Latreille, 1804

http://species-id.net/wiki/Dryinus

#### Diagnosis.

Female: macropterous; mandible with 1–4 teeth; occipital carina complete, or incomplete, or absent; antenna without tufts of long hairs on segments 5–10, usually with rhinaria, occasionally without; antennal segment 3 less than five times as long as segment 2; occasionally antennal segment 3 more than five times as long as segment 2 (in this case, notauli occasionally complete and scutum completely sculptured by numerous and parallel longitudinal keels); palpal formula 6/3; pronotal tubercle reaching or not tegula; forewing with three cells enclosed by pigmented veins (costal, median and submedian); protarsus chelate; chela with rudimentary claw; segment 5 of protarsus less than twice as broad as enlarged claw; enlarged claw as long as, or shorter than protibia; tibial spurs 1/1/2, rarely 1/1/1. Male: macropterous; mandible with 1–3 teeth; palpal formula 6/3; occipital carina complete or incomplete; lateral regions of prothorax not continuous with mesopleura; epicnemium visible; mesosternum fused with mesopleura and not distinct; forewing with three cells enclosed by pigmented veins (costal, median and submedian); paramere without dorsal process; tibial spurs 1/1/2.

#### Distribution.

Worldwide.

#### Hosts.

Acanaloniidae, Cixiidae, Dictyopharidae, Flatidae, Fulgoridae, Issidae, Lophopidae, Ricaniidae, Tropiduchidae (Guglielmino and Olmi 1997, 2006, 2007)

#### Species.

Two hundred and seventy-nine.

#### Remarks.

The Neotropical species of *Dryinus* are divided into four groups, according to the following key (Olmi 1993):

**Table:** 

1	Enlarged claw very reduced, approximately as long or slightly longer than arolium	*autumnalis* group
–	Enlarged claw not reduced, much longer than arolium	2
2	Enlarged claw without subapical tooth, or with at least 2 subapical teeth; rarely with one only subapical tooth, but then with a very broad apical lamella	*lamellatus* group
–	Enlarged claw with 1 subapical tooth, never with a broad apical lamella	3
3	Notauli at least partly present	*constans* group
–	Notauli absent	*ruficauda* group

#### Key to fossil species of the *lamellatus* group

**Females (males unknown)**

**Table:** 

1	Enlarged claw not spatulate (Figs 1, 2)	*Dryinus grimaldii* Olmi
–	Enlarged claw spatulate (Fig. 4)	*Dryinus rasnitsyni* sp. n.

### Dryinus grimaldii

Olmi

http://species-id.net/wiki/Dryinus_grimaldii

Figs 1 [Fig F2] –3

Dryinus grimaldii Olmi 1995: 254.
Dryinus grimaldii Olmi: Olmi 2000: 65.
Dryinus grimaldii Olmi: Olmi and Bechly 2001: 45.

#### Type material.

*Holotype*, female, Early Miocene amber from the Dominican Republic (16–19 Ma) (AMNH, No. DR-10-1426); same locality label, 1 female paratype (AMNH, No. DR-10-1423).

#### Additional specimens examined.

same locality, three female specimens (GPJC).

#### Diagnosis.

**Figure 1.**
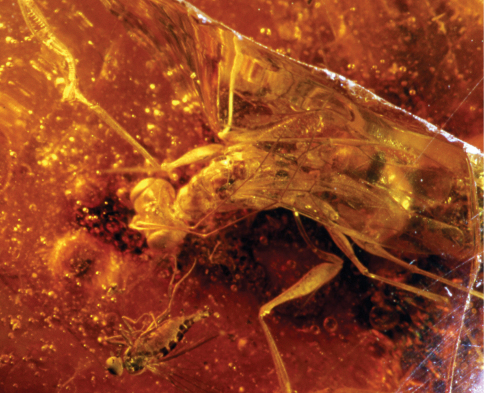
*Dryinus grimaldii*. Female holotype (from Olmi 1995). Length 4.3 mm.

**Figure 2. F2:**
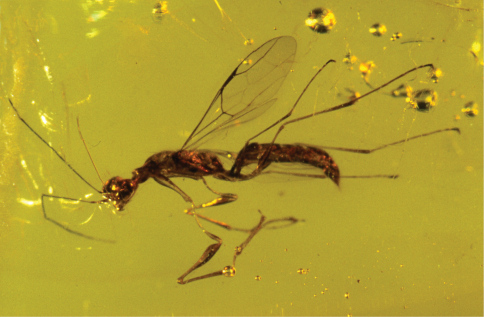
*Dryinus grimaldii*. Female specimen in lateral view (in GPJC, No. H-10-100). Length 6.3 mm.

Female with enlarged claw not reduced and not spatulate (Figs 1, 2), longer than arolium; enlarged claw with two subapical teeth. Male unknown.

#### Redescription.

*Female*: macropterous; length 4.3–6.3 mm. Colour difficult to discern, apparently testaceous, except two dark lateral spots on sides of pronotal disc, scutum, scutellum, propodeum and tegula dark; metasoma with dark transverse band. In paratype, legs with dark spots on coxae and clubs of femora; scutum apparently without dark lateral spots. In one specimen of GPJC labelled H-10-100, apparently scutum without lateral dark spots, scutellum not darkened, posterior surface of propodeum darkened. In two specimens of GPJC labelled H-10-23C, body totally testaceous, except petiole black and two brown spots on sides of scutum. Antenna 10-segmented, long and very slender, filiform, not thickened distally, covered with dense and short hairs; antennal segments of holotype in following proportions: 10:5:44:57:38:21:9:9:9:17; antenna more than nine times as long as head (length of head dorsally measured from occipital carina behind ocelli to distal apex of mandible): 219:22. Head weakly convex, apparently shiny, finely punctate, without apparent sculpture among punctures; clypeus and mandible not distinct; occipital carina apparently complete; occiput deeply excavated; eye normally bulging; POL = 2; OL = 1.5; OOL = 10; OPL = 1.5; TL = 5; greatest breadth of posterior ocellus longer than POL (4:2); frontal line absent. Maxillary palpi not evident, apparently 6-segmented. Labial palpi not distinct. Pronotum apparently shiny, finely punctate, about as long as head, crossed by anterior strong transverse impression between anterior collar and disc; disc humped; posterior collar very short; pronotal tubercle reaching tegula. Scutum apparently shiny, finely punctate, slightly shorter than pronotum (19:22). Notauli complete, posteriorly separated; minimum distance between notauli about as long as greatest breadth of posterior ocelli. In one specimen of GPJC labelled H-10-100, notauli apparently almost complete, not reaching posterior margin of scutum. Scutellum apparently shorter than scutum (10:19), with sculpture not evident. Metanotum shorter than scutellum (6:10), with sculpture not evident. Propodeum longer than scutum (39:19), reticulate rugose, areolae very broad; posterior surface with two complete longitudinal keels; sculpture of median area of posterior surface not evident. In one specimen of GPJC labelled H-10-100, dorsal surface of propodeum with two median longitudinal and almost parallel keels. Shape of pronotum, scutum, scutellum, metanotum and propodeum usual for Dryininae. Forewing hyaline, without dark transverse bands, with usual venation of Dryininae; pterostigma narrow, much longer than broad (32:4); marginal cell apparently open; distal part of stigmal vein longer than proximal part (16:11); stigmal vein not S-shaped, forming angle between proximal and distal parts; forewing with usual three basal cells clearly enclosed by pigmented veins (costal, median and submedian cells). Hindwing hyaline, without dark transverse bands. Foreleg segments in following proportions: 55 (coxa): 53 (trochanter): 61 (femur): 60 (tibia): 18 (tarsal segment 1): 5 (tarsal segment 2): 8 (tarsal segment 3): 55 (tarsal segment 4): 78 (tarsal segment 5); foreleg chelate; enlarged claw much shorter than segment 5 of protarsus (42:78); protrochanter more than four times as long as broad (53:5)(greatest breadth measured on distal club), with long and slender proximal stalk, broadened after half-way; segments 2 and 3 of protarsus produced into hooks; rudimentary claw present; arolium much shorter than enlarged claw (8:42); enlarged claw with two strong subapical teeth and 1 row of 8 lamellae; subapical teeth of enlarged claw very strong, such as in *Plesiodryinus*; distal apex of enlarged claw not spatulate. Segment 5 of protarsus with 2 rows of approximately 50 lamellae; distal apex with group of at least 20 lamellae (number of lamellae not evident). Midleg segments in following proportions: 22 (coxa): 7 (trochanter): 41 (femur): 60 (tibia): 17 (tarsal segment 1): 15 (tarsal segment 2): 15 (tarsal segment 3): 14 (tarsal segment 4); segment 5 of mesotarsus not distinct. Hindleg segments in following proportions: 27 (coxa): 11 (trochanter): 48 (femur): 80 (tibia); segments of metatarsus not distinct in holotype; segments of metatarsus of paratype in following proportions: 37 (tarsal segment 1): 19 (tarsal segment 2): 13 (tarsal segment 3): 10 (tarsal segment 4): 11 (tarsal segment 5). Metasoma without distinct and slender petiole. Shape and length of petiole usual for Dryininae. Shape, length and breadth of wings usual for Dryininae. Shape of body usual for Dryininae. Tibial spurs of holotype hardly visible, apparently 1/1/1; in one specimen of GPJC labelled H-10-100, tibial spurs distinctly 1/1/2.

*Male*: unknown.

**Figure 3.**
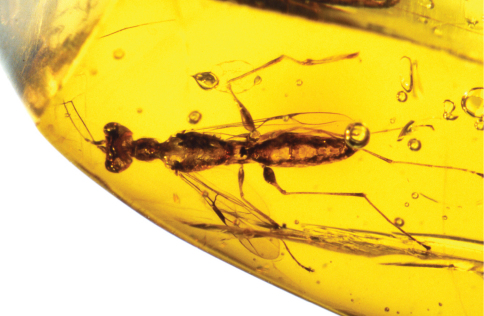
*Dryinus grimaldii*. Female specimen in dorsal view (in GPJC, No. H-10-100). Length 6.3 mm.

#### Hosts.

Unknown.

### Dryinus rasnitsyni

Olmi & Guglielmino sp. n.

urn:lsid:zoobank.org:act:5D66A0EE-F5D5-4025-878B-DD6F457E274B

http://species-id.net/wiki/Dryinus_rasnitsyni

Figs 4 [Fig F5] –6

#### Holotype.

Female, Oligo-Miocene amber from Dominican Republic (15–40 mybp)(SMSN).

#### Diagnosis.

Female with enlarged claw spatulate, not reduced, with large distal apex (Fig. 6), longer than arolium. Male unknown.

#### Description.

**Figure 4.**
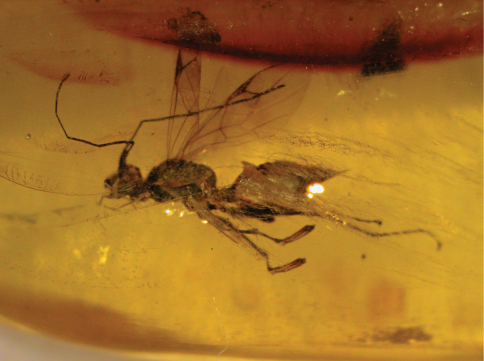
*Dryinus rasnitsyni*. Female holotype. Length 7.4 mm.

**Figure 5. F5:**
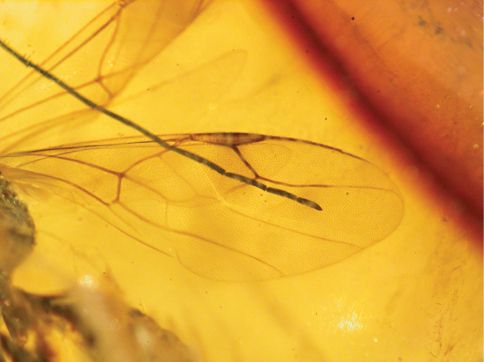
*Dryinus rasnitsyni*. Female holotype. Forewing.

**Figure 6.**
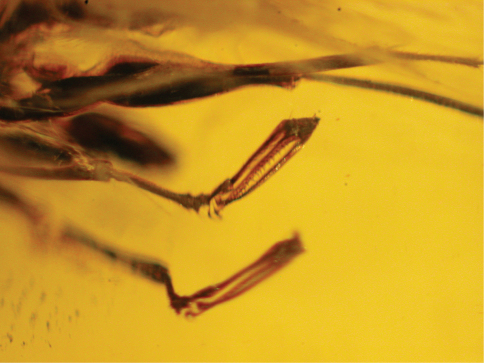
*Dryinus rasnitsyni*. Female holotype. Forelegs.

*Female*: macropterous; length 7.4 mm. Colour not distinct, apparently brown, except head, palpi and chela partly testaceous. Antenna 10-segmented, long and very slender, weakly thickened distally, covered with dense and short hairs; antennal segments in following proportions: 20:8:28:27:35:39:28:19:15:13; antenna about five times as long as head (length of head dorsally measured from occipital carina behind ocelli to distal apex of mandible): 90:18. Head weakly convex, apparently dull, granulated; occipital carina and occiput not distinct; eye normally bulging; frontal line not evident. Palpal formula apparently 6/3. Pronotum apparently shorter than head (8:18), crossed by anterior strong transverse impression between anterior collar and disc; disc humped; sculpture, posterior collar and pronotal tubercle not distinct. Scutum apparently slightly longer than pronotum (9:8), with sculpture and notauli not distinct. Scutellum apparently shorter than scutum (4:9), with sculpture not distinct. Metanotum about as long as scutellum, with sculpture not distinct. Propodeum longer than scutum (15:9), with lateral regions reticulate rugose, with dorsal surface longer than posterior surface (10:5); sculpture of rest of propodeum and posterior surface not distinct. Shape of pronotum, scutum, scutellum, metanotum and propodeum apparently usual for Dryininae. Forewing (Figs 4, 5) completely weakly darkened, with usual venation of Dryininae; pterostigma narrow, much longer than broad (36:7); marginal cell open; distal part of stigmal vein longer than proximal part (34:18); stigmal vein very weakly S-shaped, forming angle between proximal and distal parts; forewing with usual three basal cells clearly enclosed by pigmented veins (costal, median and submedian cells). Hindwing completely weakly darkened. Foreleg segments in following proportions: 29 (coxa): trochanter not visible: 57 (femur): 46 (tibia): 27 (tarsal segment 1): 5 (tarsal segment 2): 8 (tarsal segment 3): 26 (tarsal segment 4): 46 (tarsal segment 5); foreleg chelate; enlarged claw slightly shorter than segment 5 of protarsus (42:46); protrochanter not distinct; segments 2 and 3 of protarsus produced into hooks; rudimentary claw present; arolium much shorter than enlarged claw (6:42). Enlarged claw (Fig. 6) spatulate, with large distal apex. Segment 5 of protarsus apparently with 1 or 2 rows of proximal and medial lamellae (number of lamellae not distinct); distal apex with a group of few lamellae (number of lamellae not distinct). Midleg segments in following proportions: 18 (coxa): 10 (trochanter): 62 (femur): 70 (tibia): 31 (tarsal segment 1): 13 (tarsal segment 2): 9 (tarsal segment 3): 4 (tarsal segment 4): 8 (tarsal segment 5). Hindleg segments in following proportions: 20 (coxa): 12 (trochanter): 87 (femur): 88 (tibia): 36 (tarsal segment 1): 16 (tarsal segment 2): 12 (tarsal segment 3): 7 (tarsal segment 4): 13 (tarsal segment 5). Metasoma with a short petiole. Shape and length of petiole usual for Dryininae. Shape, length and breadth of wings usual for Dryininae. Shape of body usual for Dryininae. Tibial spurs 1/1/2.

*Male*: unknown.

#### Etymology.

The species is named after Dr. Alex Rasnitsyn.

#### Hosts.

Unknown.

#### Remarks.

In the holotype the head is partly crushed; the clypeus and mandible are only partly visible in lateral view so that it is not possible to count the number of teeth of the mandible and to see if the anterior margin of the clypeus is rounded or bidentate; the ocelli are only partly visible in lateral view and it is not possible to measure POL, OL, OOL and OPL; the temple is not distinct; the pronotum is only partly visible because of crushing; the scutum, scutellum, metanotum and propodeum are only visible in lateral view; both chelae are closed, so that it is not possible to see if the enlarged claw has lamellae and teeth.

## Supplementary Material

XML Treatment for Dryinus

XML Treatment for Dryinus grimaldii

XML Treatment for Dryinus rasnitsyni
